# Supplementary material for: Saccharomyces boulardii (CNCM I-745) improves intestinal damage in sepsis by remodeling intestinal flora structure
Source: Front Cell Infect Microbiol. 2025 Jul 31;15:1602792. doi: 10.3389/fcimb.2025.1602792 (PMC12350329; doi:10.3389/fcimb.2025.1602792)
Supplement: Supplementary file 1 [file DataSheet1.zip › summary/summary/7_Differential_analysis/2_LEfSe_result/Group_CW4_vs_CW0_0.05_3.0/LEfSe_Group_CW4_vs_CW0.barplot.pdf]

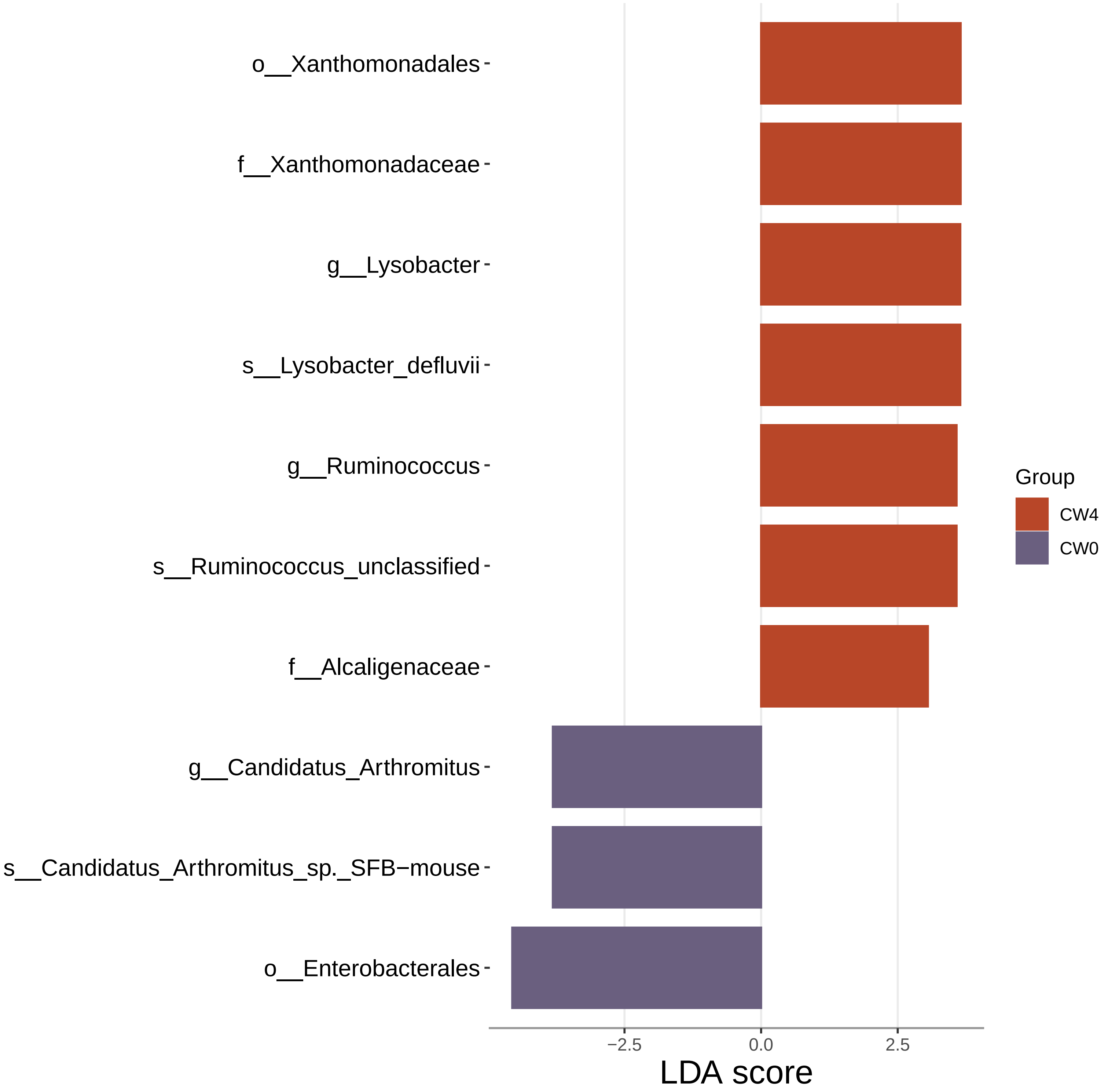
o__Xanthomonadales
f__Xanthomonadaceae
g__Lysobacter
s__Lysobacter_defluvii
g__Ruminococcus
s__Ruminococcus_unclassified
f__Alcaligenaceae
g__Candidatus_Arthromitus
s__Candidatus_Arthromitus_sp._SFB−mouse
o__Enterobacterales
−2.5
0.0
2.5
LDA score
Group
CW4
CW0
